# New insights into microvascular injury to inform enhanced diagnostics and therapeutics for severe malaria

**DOI:** 10.1080/21505594.2019.1696621

**Published:** 2019-11-27

**Authors:** Clara Erice, Kevin C Kain

**Affiliations:** a Sandra-Rotman Centre for Global Health, Toronto General Research Institute, University Health Network-Toronto General Hospital, Toronto, Ontario, Canada; b Tropical Disease Unit, Division of Infectious Diseases, Department of Medicine, University of Toronto, Toronto, Canada

**Keywords:** Severe malaria (SM), host innate response, severity markers, adjunctive therapy, microvascular injury, risk-stratification

## Abstract

Severe malaria (SM) has high mortality and morbidity rates despite treatment with potent antimalarials. Disease onset and outcome is dependent upon both parasite and host factors. Infected erythrocytes bind to host endothelium contributing to microvascular occlusion and dysregulated inflammatory and immune host responses, resulting in endothelial activation and microvascular damage. This review focuses on the mechanisms of host endothelial and microvascular injury. Only a small percentage of malaria infections (≤1%) progress to SM. Early recognition and treatment of SM can improve outcome, but we lack triage tools to identify SM early in the course of infection. Current point-of-care pathogen-based rapid diagnostic tests do not address this critical barrier. Immune and endothelial activation have been implicated in the pathobiology of SM. We hypothesize that measuring circulating mediators of these pathways at first clinical presentation will enable early triage and treatment of SM. Moreover, that host-based interventions that modulate these pathways will stabilize the microvasculature and improve clinical outcome over that of antimalarial therapy alone.

## Introduction

**Figure 1.**
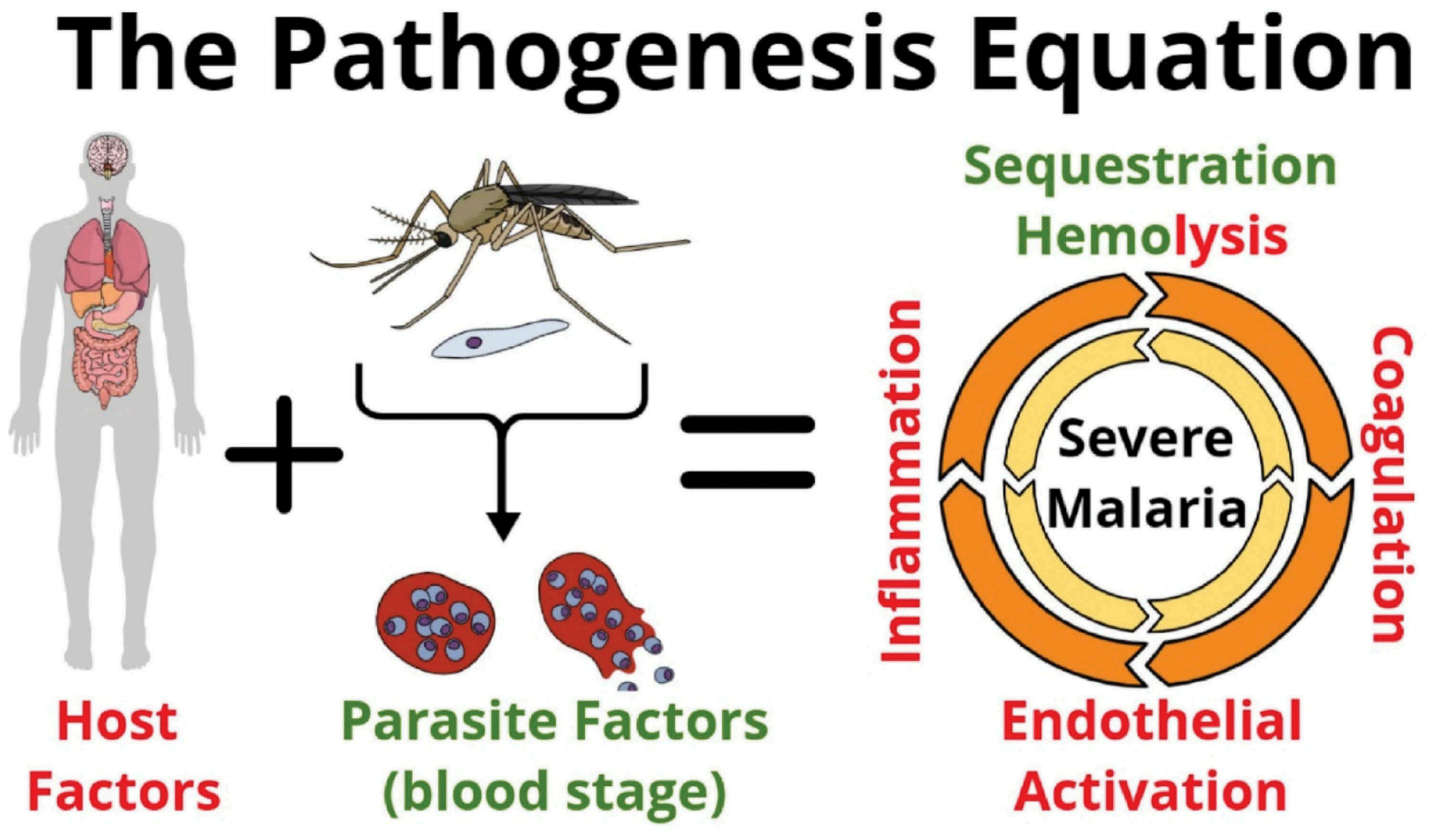
Severe malaria pathogenesis: Both host and parasite factors contribute to the pathogenesis of severe malaria (SM). Key mechanisms include, sequestration of infected erythrocytes in the microvasculature of vital organs, hemolysis, inflammation, coagulopathy, endothelial activation, and microvascular injury. Each of these mechanisms may amplify another, leading to multi-organ failure and death.

Severe malaria (SM) is a complex multi-system disorder. Although, large knowledge gaps still exist in our understanding of its pathobiology, there is a consensus that the onset and outcome of SM depends on a complex interaction between both pathogen and host determinants [1]. The SM pathogenesis paradigm argues that the combination of microvascular sequestration of infected erythrocytes (IE), together with infection-induced; immune and endothelial activation, dysregulation of coagulation cascades and microvascular occlusion and injury, ultimately culminates in the breakdown of endothelial barriers including the blood–brain barrier (BBB), multi-organ dysfunction and death [2] (Figure 1).

It is important to note that the majority of malaria infections are asymptomatic, followed by uncomplicated malaria (UM) cases, where patients manifest mild symptoms such as fever, and that only a small proportion of malaria infections (<1%) progress to SM. However, despite malaria control and elimination efforts, SM still accounted for an estimated 450,000 deaths in 2017 [3]. Even with potent antimalarial treatment, there has not been a substantial decrease in mortality rates associated with SM in children in the past decade [4]. Moreover, it is estimated that mortality rates with parenteral artesunate treatment generally range between 8.5% and ~18% [5]. More than 93% of SM cases occur in sub-Saharan Africa, where children under 5 are those most at risk [3]. *Plasmodium falciparum (P. falciparum)* is the primary cause of SM, but recent evidence indicates that *Plasmodium vivax (P. vivax)* and *Plasmodium knowlesi (P. knowlesi)* infections can also result in SM [6–8].

SM in children is generally defined as the presence of *P. falciparum* via a positive blood smear, PCR or a positive malaria rapid diagnostic test (mRDT), together with one or more of the following clinical symptoms: impaired consciousness, coma, respiratory distress, multiple convulsions, prostration, shock, pulmonary edema, abnormal bleeding, jaundice, severe anemia, hypoglycemia, acidosis, hyperlactatemia, renal impairment, and/or hyperparasitemia [9,10]. However, SM usually presents as one or more of the following overlapping syndromes; severe malarial anemia (SMA), cerebral malaria (CM), and/or respiratory distress (RD), with the highest mortality rate observed with CM and RD [11]. Both SMA and CM are associated with long-term complications [9]. In prospective studies 50% or more of children surviving CM develop neurodevelopmental and neurocognitive impairment, lasting 1 year or more after the resolution of infection. Retrospective studies suggest these deficits persist for at least 8 years [9,12–14]. SMA has also been associated with over-all impaired cognitive ability [15], indicating that SM-related morbidity may continue long after successful clearance of the parasite.

Although mRDTs have transformed malaria diagnosis in many low and middle-income countries (LMICs), it is important to emphasize that they do not inform critical management decisions including whether a patient has, or is progressing to, SM, and if therefore, needs a referral, admission, and intravenously administered artesunate. National surveys, carried out in sub-Saharan Africa, indicate that 10% or less of malaria cases are appropriately triaged for care. Moreover, when a child presents to an emergency department with SM, less than 30% are diagnosed and treated promptly, resulting in increased mortality and neurocognitive deficits in survivors [16–18]. Early recognition and treatment of SM can save lives and prevent brain injury, however, we currently lack rapid and accurate triage tools for SM.

In the following sections, we will review the pathogen and host factors contributing to SM, and explore whether these can be exploited to improve the early recognition and triage of SM, with a specific focus on host-factors. For parasite determinants, we will briefly discuss the asexual blood stage of the infection which is responsible for the manifestations of SM. Targeting earlier stages in the life cycle, such as the liver stage, also represents an encouraging area for investigation. For an excellent review on these possibilities please refer to [19]. Recent evidence also supports a role for host-factors not only in contributing to SM, but also supporting the clinical utility of measuring biomarkers of these pathways as accurate community-based prognostic tools to triage children with malaria, as well as, intervention points for adjunctive therapies to attempt to improve clinical outcome [20,21]. Moreover, since multiple severe infections (e.g. sepsis) appear to share similar pathways of host-response and microvascular injury, as SM, we explore the hypothesis, that measuring host markers of immune and endothelial activation at clinical presentation, may allow to risk-stratify febrile syndromes irrespective of etiology. This approach could enable integrated and evidence-based point-of-care (POC) decision-making for “all cause” fever syndromes in low-resource settings [20,22].

## P. falciparum and severe malaria: Cytoadherence and parasite biomass

One of the key events during SM pathogenesis is the capacity of IE to cytoadhere to endothelial cells lining the microvasculature of vital organs, for example, the brain in CM [23]. This allows IE to sequester and avoid clearance by spleen and liver macrophages [24]. IE express variants of the parasite protein, *P. falciparum* erythrocyte membrane protein 1 (PfEMP1), on their cell surface. These proteins, encoded by highly variable var genes, are able to bind to multiple cell adhesion molecules on endothelial cells including: intracellular cell adhesion molecule 1 (ICAM-1), CD36 and endothelial protein C receptor (EPCR) [25]. IE cytoadherence promotes a dysregulated host response cycle, as pro-inflammatory cytokines and chemokines, triggered by IE, will upregulate the expression of cell adhesion molecules, allowing for more sequestration to occur. This contributes to vascular occlusion, metabolic acidosis, enhanced inflammation, coagulopathy and endothelial activation resulting in microvascular damage (Figure 1) [26].

Quantitative measurements of plasma levels of the malarial protein, histidine-rich protein 2 (HRP2), have shown to predict progression to CM [27]. A recent meta-analysis reports that parasite biomass, as determined by parasitemia and quantitiative plasma HRP2 levels, and parasite var gene profiles are predictors of progression to SM in both adults and children [28]. Furthermore, *in vitro* findings using patient parasite isolates, support the theory that there is an association between parasite binding affinity to specific endothelial receptors in different tissues and disease pathology [29]. The seminal discovery that some PfEMP1 variants could bind to EPCR, linked sequestration events directly with host-coagulation, inflammation and endothelial activation (reviewed in [30]). In accordance with this, PfEMP1 subtypes that bind EPCR, have been linked to brain swelling in pediatric CM [31,32]. IE not only have the ability to bind to endothelial cells but are also able to bind uninfected erythrocytes, producing rosettes [33,34]. Rosetting is not only mediated by PfEMP1. The surface variant proteins subtelomeric variant open reading frame (STEVOR) and repetitive interspersed repeats (RIFIN), can also bind to uninfected erythrocytes [35]. Rosettes have been linked to all presentations of SM in African children [36]. IE can also form clumps by binding to platelets [37], which has also been associated with SM, in particular, CM [38]. These events may contribute to microvascular damage by increasing vascular obstruction (reviewed in [1]).

De-sequestration strategies have been explored as therapeutic approaches to reduce cytoadherence and rosetting. Considerable advances are being made in understanding parasite binding specificities, and how to exploit these for diagnosis and treatment. However, de-sequestration interventions would rely on receptor blockade, generally involving the use of recombinant proteins or antibody blockade. These approaches are challenging both in identifying who would benefit, especially early enough in the course of illness, and in how to administer in a low resource-setting, due to their cost and potential storage requirements [19]. We direct the reader to more in-depth reviews looking at cytoadherence adjunctive therapies [2,19]. Since parasite biomass is also a predictor of progression to SM [28], whole blood transfusions and automated erythrocyte exchange, to decrease parasitemia, have been used as putative adjunctive therapies, although no randomized clinical trials have confirmed their superiority over the standard of care (reviewed in [39]). Additionally, due to cost and availability, these strategies are often untenable in most low-resource settings [2]. If parasite phenotypes are reproducibly found to predict progression to SM, then parasite isolate genotyping could be exploited to risk-stratify malaria patients [28]. However, technical and financial constraints associated with genotyping at point-of-care in low-resource settings make this an unattractive approach at present. Therefore, although, cytoadherence is a promising area, additional research is needed to demonstrate that this would be an accurate and efficient diagnostic or prognostic tool, or an efficacious adjunctive therapeutic approach. Importantly, both sequestration and rosetting impact the host innate response, at the level of the endothelium, by inducing pro-coagulant pathways as well as dysregulating inflammation and endothelial function, which will be covered in the next sections.

## Host factors and microvascular injury

### Coagulation and inflammation

The host innate immune response plays a critical role in the initial recognition and clearance of invading pathogens including malaria. Innate responses are tightly regulated in order to avoid immunopathology, however, malaria infection may dysregulate these response pathways and contribute to disease severity and host survival [40]. The role of the innate immune response in driving SM pathogenesis is well documented [41], in particular, dysregulation of the coagulation cascade [42]. Furthermore, there is cross-talk between the pathways that mediate inflammation and the coagulation cascade [43]. The complement system and coagulation share an evolutionary link and both influence the host innate response, thus, giving rise to the concept of “immuno-thrombosis” [44]. Tissue Factor (TF), which initiates the clotting cascade when activated by Factor VIIa, is considered to be at the interface of inflammation and coagulation [45]. In line with this, and in the context of malaria, it has been reported that IE trigger TF expression on endothelial cells *in vitro*, which in turn supports the aggregation of coagulation complexes. Moreover, the same group showed that children who died from CM have positive immunohistochemical staining for TF on their brain microvasculature [46]. Coagulation factors are pro-inflammatory, driving the expression of cytokines, which further induce TF expression. Additionally, IE binding to EPCR will inhibit activated protein C (APC), resulting in higher levels of activated coagulatory Factor VIIIa. In combination all these events exacerbate a pro-coagulatory and pro-inflammatory state, contributing to the cycle of SM pathogenesis (Figure 1) [45]. We direct the reader to specific reviews on SM pathogenesis and coagulation [30,42]. This review focuses on inflammation and particularly how the complement system may initiate and amplify microvascular damage during disease.

The complement cascade is a core component of innate immunity. There are three major pathways of the complement system (classical, lectin and alternative), all converging on C3, which subsequently gives rise to the activation products C3a, C3b, and downstream C5a. C5 can also be directly cleaved to the active anaphylatoxin C5a both by, thrombin and serine proteases released by activated neutrophils and macrophages, as well as by malaria parasites [47,48]. Different components of the complement system are activated in populations progressing to SM [47,49,50]. During malaria infection, free hemoglobin (Hb) and heme released during IE rupture, can directly activate C3 on the surface of endothelial cells (see section 3.3) [51]. C5a, generated by the above routes, can markedly potentiate the induction of pro-inflammatory cytokines *in vitro*, which have been associated with SM [48,52]. Parasite toxins from ruptured IE, such as glycosylphosphatidylinositol (GPI), can bind Toll-like receptors (TLR), which activate the transcription of inflammatory cytokines via the NF-κB pathway [11]. Furthermore, during SM there is an increase in the production of immune cells, such as macrophages and neutrophils which in turn produce cytokines as well [53]. Elevated levels of TNF and IL6 cytokines are classically associated with severe *P. falciparum* malaria and have been proposed as markers of CM [54]. However, no consensus on a distinct pattern of cytokines predicting severity has been reached, as there are contradicting findings, hampering the ability to designate specific cytokine profiles as biomarkers (reviewed in [55]). What is clear is that complement activation enhances inflammation, endothelial activation, and coagulation, making it an early target that initiates and augments many aspects of SM pathogenesis [47].

In accordance with this, a recent proteomic analysis, showed that there was an increase in proteins in biological pathways involved in the innate immune response and complement system in CM patients [56]. Moreover, a pilot study looking at microarray data from a population from Mali with SM and UM showed that differential and increased expression of TLR and complement proteins was associated with disease severity [57]. Circulating levels of C5a in children with CM were reported to be higher than those of children with UM. These observations were explored in a murine model of CM, which showed that C5a and its receptor, C5aR1, contributed directly to experimental cerebral malaria (ECM) pathobiology. Mice not expressing C5aR1 had decreased inflammation and endothelial activation [58]. These findings were supported by other studies, showing a role for C5a and C5aR1 and complement activation, and their relationship to disease severity, in particular increased levels of IL8/CXCL8 [59]. Additionally, children with SMA also have dysregulated complement activation. Specifically, links between decreased levels of complement regulatory protein 1 (CR1), leading to increased uninfected erythrocyte clearance and increased anemia, which has been proposed as a pathobiological pathway. Increased levels of cleaved complement elements have also been associated with children with SMA [60,61]. However, there is some controversy since another investigation found no link to SMA and complement activation, but did see a reduction in complement regulatory proteins [62]. It has been proposed that excessive production of pro-inflammatory cytokines, such as IFNγ and TNF, contribute to the pathobiology of SMA by dysregulating erythrocyte production and increasing erythrophagocytosis [63]. Malaria during pregnancy (MiP) is outside the scope of this review. Nonetheless, it is important to highlight that the complement system, specifically C5a, plays a crucial role in its pathogenesis [48,64], reviewed in [65]. We direct the reader to in-depth reviews on the complement system and innate immunity and malaria pathobiology [41,47,63].

In summary, uncontrolled complement activation and its ability to potentiate inflammation ultimately contribute to endothelial activation and microvascular injury.

### Endothelial activation

Markers of endothelial activation measured at clinical presentation have been shown to be independent and quantitative predictors of malarial disease severity and outcome [66–72]. Clinical trials and retrospective case–control studies, in pediatric malaria in sub-Saharan Africa, have linked markers of endothelial activation with microvascular thrombosis, coma duration, and neurocognitive outcome, as well as with, multi-organ dysfunction, acute kidney injury (AKI) and respiratory distress [21,66,73–76]. Endothelial activation is highly interconnected with coagulation, immune activation, and inflammation. During endothelial quiescence or homeostasis, endothelial cells express anti-coagulant factors such as APC and thrombomodulin. However, when triggered by the binding of IE (especially to EPCR), and platelets or the complement activation, endothelial cells undergo a switch toward a pro-coagulatory and inflammatory state (discussed in 3.1). This is nicely highlighted by an *in vitro* study, which used human brain endothelial cells and exposed them to IE, to later analyze their global gene expression. They reported increases in the expression of transcripts involved in the immune and inflammatory response [77]. Accordingly, other groups have also reported increased expression of TF, von Willebrand factor (vWF), and cell adhesion molecules (e.g. ICAM-1, VCAM and E-selectin), reviewed in [78].

An integral component of endothelial quiescence and vascular stability is the Angiopoietin (Ang) and Tie axis. Ang-1 is expressed by pericytes, mural cells, and platelets. Under normal physiological conditions Ang-1 binds to its cognate receptor Tie-2 on endothelial cells promoting tight junction (TJ) formation and endothelial stability. Moreover, Ang-1/Tie-2 signaling favors an anti-inflammatory and anti-apoptotic state. However, Ang-2 is also a ligand for Tie-2, but is an antagonist to endothelial stability, and drives a pro-inflammatory and pro-thrombotic environment and microvascular destabilization. In a non-disease state, there is a higher concentration of circulating Ang-1 than Ang-2 promoting microvascular quiescence. During malaria infection, Ang-2 and vWF are released from Weibel-Palade bodies (WPB) within the endothelial cells, antagonizing Tie-2 activation and triggering a pro-coagulant and pro-inflammatory state, associated with microvascular activation and a risk of SM, reviewed in [79]. Hence, the ratios of Ang-1 and Ang-2 are indicative of endothelial dysregulation, where higher Ang-2 and lower Ang-1 levels indicate progression to severe and potential fatal disease [80]. For example, circulating levels of endothelial activation markers such as sICAM-1, vWF, Ang-2 and sTie-2 are associated with retinopathy, CM and death – linking IE sequestration, endothelial activation, vascular injury and clinical outcome [74].

Other pathways regulating microvascular function, such as Slit-2/Roundabout (Robo4), may also be involved in the pathobiology of SM. Robo4 is expressed by endothelial cells, and when activated via Slit-2, it promotes endothelial quiescence by blocking Src-mediated VE-cadherin internalization, a key component of adherens junctions [81–83]. Consistent with this role, lower Slit-2 expression is associated with microvascular dysfunction and severe infections [83,84]. Moreover, the addition of synthetic Slit-2 has been shown to improve survival and vascular stability in disease models of sepsis and severe influenza [85]. A role for the Slit-2/Robo4 pathway has not yet been reported in SM pathogenesis; however, this pathway represents an attractive area to pursue and identify putative host-targeted therapeutics.

**Figure 2.**
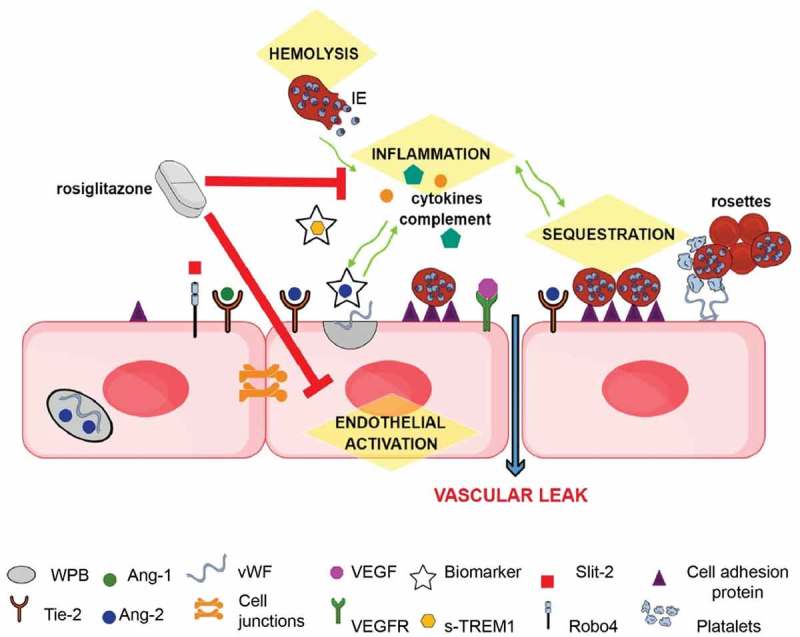
Schematic of mechanisms underlying SM pathology that represent potential sites for therapeutic intervention (highlighted in yellow): During quiescence, Ang-1 via Tie-2, and possibly Slit-2 via Robo4, promote endothelial integrity by stabilizing junctional proteins. During severe infections Weibel-Palade bodies (WPB) in the endothelial cell, stimulated by inflammatory responses, release Ang-2 and vWF. Ang-2 displaces Ang-1 and inhibits Tie-2 endothelial stabilizing effects. The expression of cell adhesion molecules, such as ICAM-1, are increased, leading to enhanced sequestration, and vWF released from WPB, promoting a pro-coagulatory state. These pathological mechanisms may interact and exacerbate each other (green arrows), leading to microvascular dysfunction. Circulating levels of Ang-2 and sTREM-1 are good prognostic markers of disease severity and outcome. Rosiglitazone is a promising putative adjunctive therapeutic as it acts upon several of the pathological mechanisms linked to the development of SM.

Vascular endothelial growth factors (VEGF) and their receptors (VEGFRs) are also important mediators of endothelial stability. Unlike Tie-2 and Robo4, activation of VEGFR-2 on endothelial cells results in enhanced endothelial permeability through cell junction and cytoskeletal readjustments. Furthermore, VEGF acts synergistically with Ang-2 to promote endothelial activation and leak. VEGF promotes the expression and release of Ang-2 and the cleavage of Tie-2 from the endothelial surface (generating soluble Tie-2) and making it unable to stabilize the endothelium. Moreover, VEGF increases endothelial cell adhesion molecule expression thus enhancing IE sequestration, which in turn promotes inflammation and coagulopathy, reviewed in [86]. This highlights the highly intertwined relationship of the mediators driving SM pathogenesis and how they converge at the microvascular interface (Figure 2).

In accordance with their role in regulating endothelial activation, malarial disease severity and fatal outcomes in adults and children are associated with decreased circulating levels of Ang-1, or increased levels of Ang-2, or increased Ang-2/Ang-1 ratios [66,74,87–90]. An important causal role for the Ang/Tie-2 axis in severe malaria infections has been established using mechanistic studies in mice with ECM, where Ang-1 is essential in maintaining vascular and BBB integrity [91]. Moreover, a systematic review reported that decreased Ang-1 and increased Ang-2/Ang-1 ratios are good biomarkers to distinguish UM from CM and further, to prognosticate outcome [78]. The link between VEGF pathway and its role during SM pathogenesis is strong. However, there are some discrepancies in human studies as to the predictive value of measuring circulating VEGF [86,92]. For example, in Malawian children, VEGF is increased in CM patients and associated with retinopathy [74], and in Kenyan children with CM, high levels of circulating VEGF are associated with increased risk of seizures and intracranial pressure [93]. On the other hand, it has been reported that lower levels of VEGF are associated to CM mortality in Indian children [94]. Nonetheless, soluble FMS-like tyrosine kinase 1 (sFlt-1), which is a splice variant of VEGFR-1, is a promising candidate biomarker for SM prognosis. The mechanism by which sFlt-1 contributes to disease is unknown; however, it may bind to VEGF inhibiting endothelial permeability [21]. Importantly, increased levels of sFlt-1 have been associated to SM and fatal SMA in two independent studies [21,73].

Nitric oxide (NO) plays a critical role in mediating endothelial stability. NO is a signaling molecule which stabilizes the endothelium by promoting anti-inflammatory and anti-coagulatory pathways and by decreasing the expression of cell adhesion molecules. Specifically, it achieves this by inhibiting the release of Ang-2 from WPB and the production of TNF and the aggregation of platelets, while promoting the expression of Ang-1 (reviewed in [86]). NO is synthesized by the enzyme family of nitric oxide synthases (NOS) from L-arginine. Patients with SM have reproducibly been shown to have low NO bioavailability and to have endothelial dysfunction, both in children and adults, which is linked with hypoarginemia [95–97]. Low levels of bioavailable NO are due to several aspects of malaria pathobiology. Erythrocytes contain arginases, which degrade L-arginine following IE rupture. Moreover, erythrocyte rupture releases free hemoglobin which sequesters NO [98].

### Hemolysis and the heme axis

Malaria causes hemolysis of both healthy and IE. Intravascular hemolysis is the rupture of erythrocytes within the circulation which causes the release of free hemoglobin (Hb). Free Hb can be oxidized into free heme [99]. Importantly, free heme has been associated with complement activation, endothelial injury and SM pathogenesis [51,100,101]. During healthy physiological conditions, free Hb and heme are cleared from the circulation by haptoglobin and hemopexin, respectively, thus avoiding toxicity. Haptoglobin-Hb complexes are scavenged and endocytosed by macrophages, which triggers the expression of heme-oxygenase-1 (HO-1). HO-1 is protective as it breakdowns free heme. As a backup, hemopexin is able to directly bind free heme and clear it from the plasma (reviewed in [102]). However, when there is extreme hemolysis, as during malaria, these protective pathways become overwhelmed leading to deleterious amounts of free Hb and heme. This, in turn, triggers a number of pathways associated with endothelial dysfunction and SM. These events include the promotion of a pro-coagulatory environment, leukocyte activation and migration, increased expression of cell adhesion molecules, activation of the complement system and inflammation and exocytosis of WPB and thus Ang-2 and vWF. Moreover, free Hb is a scavenger of NO, further exacerbating endothelial activation by reducing the bioavailability of NO [51,103–108]. Therefore, hemolysis contributes to SM pathogenesis by a number of pathways that ultimately result in vascular destabilization (Figure 2).

A key finding suggesting that the heme axis was potentially involved in the pathogenesis of SM, was the observation that mice with ECM had significantly higher levels of free heme compared to BALB-c mice, which are resistant to ECM. Furthermore, when BALB-c mice were injected with heme and ECM, these mice died compared to “infected” ECM-resistant controls with no injected heme. Additionally, they reported that carbon monoxide (CO) offered protection against the onset of ECM. CO is produced when HO-1 catabolizes free heme, and in turn, CO can bind to free Hb in a positive beneficial autoregulatory loop [109]. In accordance with this, reports on human cohorts and mechanistic studies in mice further implicated the role of heme in the pathogenesis of SM. For example, in a Ugandan population, children with UM had higher levels of hemopexin and lower levels of heme compared to children with SMA and CM. Additionally, increased levels of heme and free Hb, in patients with SM, was associated with AKI and mortality. The same study carried out mechanistic experiments in ECM, and was able to determine a casual role for the heme axis in SM. They showed that mice that do not express hemopexin have increased malaria-related mortality compared to controls [108]. Similarly, in Indian adults with SM higher levels of plasma heme are associated with increased levels of cytokines and chemokines and disease severity [110]. Further evidence that the dysregulation of the heme axis plays a role in the pathogenesis of SM, comes from the fact that the ratios of increased heme to hemopexin in African children, are not only associated with disease severity, but with the levels of endothelial activation biomarkers, such as Ang-2 and sTie-2 [111].

## Neurological sequelae

Taken together, the available evidence indicates that multiple pathways implicated in the pathogenesis of SM all converge at the endothelial interface resulting in microvascular injury and leak. This is particularly deleterious at the BBB, where endothelial dysfunction and leak likely contributes to neuron injury and consequent brain damage [14]. Neurocognitive deficits, seizure disorders and neurobehavioural disorders are reported to occur in up to 53% of children surviving CM [12,112,113].

Of importance, it has become apparent that proteins classically considered immune response proteins, such as cytokines, chemokines, and complement proteins are also key players during normal nervous system development. For example, the complement system has been linked to synaptic pruning [114], IL6 to neurogenesis [115] and the TNF superfamily members are potent negative and positive regulators of axonal and dendritic outgrowth both in the PNS [116] and the CNS [117]. Taking into account the close association of the complement system, IL6 and TNF to SM outcomes, it is of considerable interest to consider how the essential neurodevelopment roles these proteins play during normal brain development may be altered by concomitant malaria infections during pregnancy (MiP) or in early childhood. Neurological sequelae may be the result of a combinatorial effect of not only ischemic and apoptotic events damaging neurons [14], but of dysregulated in-utero and postnatal neurodevelopment. There is a large knowledge gap in how neurodevelopment may be dysregulated and how this might contribute to neurological impairments of SM survivors and in offspring of MiP mothers [118]. Furthermore, insights into mechanisms of brain injury could highlight better adjunctive neuroprotective therapies.

## Adjunctive therapies

Collectively the existing evidence on the pathobiology of SM supports the development of adjunctive therapies that mitigate endothelial activation and microvascular injury/leak (e.g. BBB disruption) with the ultimate goal of improving survival and neurocognitive outcome. Ideally, adjunctive therapeutics would need to be initiated as early as possible in the course of disease and be able to preserve endothelial integrity in vital organs, especially the brain. This remains an operational challenge; however, one promising approach to improve the early recognition of severe malaria is through the use of better triage and risk stratification tools (e.g. severity markers, see section 6) so that antimalarial therapy and adjunctive therapies could be co-administered earlier in the course of disease [20].

Similar to sepsis, adjunctive therapies based on modulating cytokine and chemokine responses were initially explored for SM. However, for both sepsis and SM, this approach has proven to be challenging to deliver and clinically unsuccessful. Randomized clinical trials attempting to block specific cytokines, for example TNF inhibitors, have given contradictory results [2]. When tackling the inflammatory response, due to its complexity, better strategies may be to target early initiation events, or the convergence of multiple pathways that are implicated in the pathogenesis of SM, for example endothelial dysfunction. Alternatively, another strategy would be to identify interventions that act on these multiple pathways simultaneously.

Along this line, anti-C5 or anti-C5aR monoclonal antibodies or peptides to block C5a activity have been proposed as potential therapeutics. For example, Eculizumab, which is an anti-C5 therapy, has been approved as a treatment in several diseases. However, in the context of SM, it would likely need to be used early in the course of illness, requiring triage tools for early risk stratification of patients [19]. Even more daunting is the exorbitant price of this drug, making this approach unrealistic in low-resource settings [119]. Nonetheless, targeting complement activation, perhaps with less expensive peptide inhibitors of C5aR, remains a promising area. Increasing the bioavailability of NO is also of interest. Inhaled NO (iNO) has undergone randomized clinical trials, demonstrating it is safe, however, no significant differences in endothelial activation markers, the study endpoint, between arm studies was observed [120,121]. On the other hand, this randomized placebo-controlled double-blind study of iNO in Uganda, suggests that this adjunctive therapy might be neuroprotective. This would be the first adjunctive therapeutic that has been assessed for neuroprotection [122]. Similarly, the Ang-1/Tie-2 axis would be a good target due to its involvement in maintaining endothelial quiescence. The recombinant human Ang-1 protein, BowAng1, which is able to phosphorylate and activate Tie-2, improves survival and restores BBB integrity in ECM models when administered after the onset of BBB dysfunction [91]. This study showed that even after the initiation of microvascular leak as determined by Evan’s blue dye extravasation into the brain parenchyma, agents such as BowAng1, can prevent further brain injury and death compared to artesunate alone. More therapeutic interventions targeting the Ang-1/Tie-2 axis are reviewed in [79]. Likewise, the heme axis is a potential intervention point that remains to be explored. It would be of interest to start examining other pathways known to regulate endothelial quiescence and if they are involved in SM pathogenesis, and hence if they are potential intervention points (e.g the Slit-2/Robo4 pathway).

Drug repurposing is an attractive option to identify novel therapeutics for SM, bypassing the time and cost involved in drug design pipelines. Angiotensin II receptor modulators have been proposed as adjunctive therapeutics to promote endothelium integrity during CM. Angiotensin II type 1 receptor (AT1) activation promotes vascular permeability while Angiotensin II type 2 receptor (AT2) counteracts these effects. A pre-clinical study, has demonstrated that targeting either AT1 or AT2 stabilizes the endothelium after exposure to IE *in vitro* and improves survival rates for ECM when Angiotensin II receptor modulators are administered after the manifestation of neurological symptoms. AT1 inhibitors are routinely used to treat hypertension, and hence could be repurposed for SM; however, they run the risk of inducing hypotension [123]. Importantly an AT2 agonist, compound 21, circumvents this potential side effect, and has been granted orphan drug status [124]. Statins, which are a class of lipid-lowering drugs, have also been proposed as possible adjunctive therapeutics for SM. They have been shown to have both anti-inflammatory and neuroprotective effects, and atorsvastatin has antimalarial properties *in vitro*. Moreover, during ECM infection, mice treated with atorvastatin in combination with antimalarial treatment compared to mice treated with antimalarials alone, have delays in the time of death as well as in the onset of neurological symptoms and parasitemia levels [125]. Another promising adjunctive drug for the treatment of SM is the PPAR gamma agonist rosiglitazone. Rosiglitazone was initially developed to treat type II diabetes. Rosiglitazone, targets multiple pathways implicated in the pathogenesis of SM, including inflammation, oxidative stress and endothelial activation (Figure 2) [126]. Moreover, it increases neurotrophic factor expression (e.g. BDNF, NGF) and has been shown to be neuroprotective in pre-clinical models of ECM. Mice treated with rosiglitazone and artesunate, after the onset of neurological symptoms, had better neurological outcomes than mice treated with artesunate alone [127]. It has been shown to be safe as an adjunctive therapeutic in UM in adults [128] and children and now is undergoing a phase IIb clinical trial for pediatric SM [129]. We direct the reader to an in depth recent review on adjunctive therapeutics [2].

## Severity markers and risk stratification

Immune and endothelial activation, and the pathways that regulate them, have been implicated in multiple life-threatening infections including SM (reviewed in [20]). For example, dysregulation of the Ang/Tie-2 axis has been linked to disease severity and outcome in clinical studies and/or pre-clinical models for several infections including sepsis, streptococcal toxic shock syndrome, *Escherichia-coli*-related hemolytic-uremic syndrome and Ebola among others [130–136]. Although additional studies are required, the available evidence supports the hypothesis that therapies that stabilize the endothelium could improve outcomes across a broad range of severe infections. This could enable the administration of broad-spectrum therapeutics that restore microvascular stability irrespective of etiology at patient presentation [80].

This approach would ideally be linked to triage tools to risk-stratify patients most likely to benefit from these pathway-directed high-value therapeutics. Circulating mediators of immune and endothelial activation (e.g. Ang-2, sTREM-1) measured at first clinical presentation have been shown to be accurate in risk stratifying not only in malaria patients (Figure 2), but for all cause febrile syndromes [79,80,88,137,138]. Although, large prospective studies will be required to prove clinical utility, incorporating these markers in rapid POC tests could permit risk stratification of fever syndromes in both low and high resource settings.

## Concluding remarks

A detailed understanding of the molecular mechanisms that underlie SM pathogenesis can facilitate the design of effective adjunctive therapeutics (discussed in [19]). Rosiglitazone is one example of a re-purposed drug that targets multiple pathways of injury linked to malaria disease severity and death. However, the optimal efficacy of any new adjunctive therapy will be enhanced if it can be directed to patients with SM as early as possible in the disease course. Therefore, POC triage tools that permit early recognition and risk-stratification of children and adults with impending SM, could facilitate early and appropriate administration of both parenteral antimalarials and adjunctive and/or neuroprotective agents, with the goal to improve survival and reduce brain injury.
